# Intensification and intrahousehold decisions: Fertilizer adoption in Burkina Faso

**DOI:** 10.1016/j.worlddev.2017.11.012

**Published:** 2018-05

**Authors:** Hamza Haider, Melinda Smale, Veronique Theriault

**Affiliations:** a Department of Agricultural, Food, and Resource Economics, Michigan State University, 105 Cook Hall, East Lansing, MI 48824-1039, USA; b Department of Agricultural, Food, and Resource Economics, Michigan State University, Justin S. Morrill Hall of Agriculture, 446 West Circle Dr., Rm 219, East Lansing, MI 48824-1039, USA; c Department of Agricultural, Food, and Resource Economics, Michigan State University, Justin S. Morrill Hall of Agriculture, 446 West Circle Dr., Rm 213-B, East Lansing, MI 48824-1039, USA

**Keywords:** Intrahousehold, Intensification, Fertilizer, Burkina Faso, Bargaining

## Abstract

In most West African countries, agricultural production is a complex process that involves multiple household members managing land and sharing agricultural inputs. We develop an intrahousehold decision-making model to illustrate how technology adoption can influence bargaining processes on farms in harsh agricultural environments of West Africa, where staple food production by extended families is organized collectively under the leadership of a senior head. The head, who assumes primary responsibility for household food security, also allocates fields to members who manage production individually. Drawing on this and the intrinsic complementarity of labor and fertilizer as divisible inputs, we test the nature of the linkage between fertilizer use on collective and individual plots by applying bivariate probit and tobit models to nationally-representative, panel data from Burkina Faso. We find evidence of input sharing, though bargaining is inadequate to sustain efficient allocation of fertilizer. Plot manager characteristics that influence bargaining power, such as literacy, gender, age, contact with extension, and membership in farmer organizations differ between collectively- and individually-managed plots—confirming the differential status of household members in technology adoption. Agroforestry practices are strongly and positively associated with fertilizer use, regardless of plot manage type. Programs aimed at increasing use of modern agricultural inputs should consider impacts on bargaining positions within the household. Providing inputs to women and young men will increase their influence on other decisions, which may lead to greater equity within the household. It may also enhance efficiency in production, since currently, inputs are not allocated efficiently within the household.

## Highlights

• Intrahousehold bargaining affects the adoption of fertilizer on farms in West Africa.
• Overall fertilizer use rates are low for cereals but less so for maize in Burkina Faso.
• Family members share agricultural inputs, including fertilizer.
• Bargaining does not achieve efficient fertilizer allocations.
• Inclusive policy should consider who within the household has access to inputs.

## 1 Introduction

Collective organization of farm production by extended family households is a social norm in the dryland farming systems of West Africa, including most regions of Burkina Faso. Often, households that span multiple generations and encompass several nuclear families farm together under the management of a senior male head or his designate. Historically, in this harsh environment, with limited equipment and few modern inputs, family groups may have averted hunger through effective pooling of their land and labor. Chayanov, 1991, Fafchamps, 2001 and others have invoked risk and uncertainty to explain various forms of collective farming, and a recent empirical analysis by Ouedraogo (2016) supports this viewpoint for dryland production in Mali.

Today, extended family households in this region farm a mixture of collectively and individually managed fields. While individual plots proliferate, production on large collective fields continues to serve as the basis for family food security on many farms. Some researchers suggest that the head’s strategy is to encourage hard work on these fields by granting “private” plots as rewards to family members (Fafchamps, 2001, Guirkinger and Platteau, 2014). Intensification, including the adoption of modern inputs such as fertilizer, may also explain individualization of production processes as a consequence of management diseconomies (Gray and Kevane, 2001, Guirkinger and Platteau, 2014).

The unitary model of household decision-making is ill-suited to exploring technology adoption in this context. The unitary model, which assumes a single or altruistic welfare function among family members (Becker, 1981)^1^, has been challenged in development research for decades (e.g., Folbre, 1984, Haddad et al., 1994, Jones, 1983, van Koppen, 2009). Several types of models have been proposed as alternatives to the unitary model, each emphasizing heterogeneous preferences and unequal status among household members. Cooperative models, which are based on game theory, specify bargaining options that are exogenous to the household and thus amenable to policy instruments (Manser and Brown, 1980, McElroy and Horney, 1981). Collective models are a special case of cooperative models (Chiappori, 1992), in which no decision-making mechanism is specified but decisions are assumed to be Pareto efficient based on sharing rules that are empirically identifiable. Non-cooperative models provide a framework for testing Pareto efficiency (Doss, 1996).

Testing a cooperative model with 1980s data from Burkina Faso, Udry (1996) rejected Pareto-efficiency of farm production based on systematic yield differentials among plots. More recently, also in Burkina Faso, Kazianga and Wahhaj (2013) demonstrated that yields on the collective plots managed by household heads were higher than those managed individually, explaining this result by “the social institution that places a particular obligation on the head of the household” (2013: 540). Following a specification similar to Udry, 1996, Ouedraogo, 2016 found more intensive use of labor, and higher productivity, on collective as compared to individual plots in Mali. By contrast, in a higher rainfall region of Mali, Guirkinger, Platteau, and Goetghebuer (2015) concluded that plots managed by individuals had higher productivity than those managed collectively by heads, especially for cash crops.

In none of these studies did authors explicitly examine linkages between input use on collective and individual fields. Direct outcomes of intrahousehold negotiation include the allocation of modern inputs, like fertilizer, among household members. Yet, intrahousehold bargaining models are largely absent from the literature on technology adoption (Doss, 2013). One noteworthy exception is the work by Von Braun and Webb (1989), who concluded that the introduction of centralized pump irrigation in the Gambia led to a transfer of the rice crop from women’s individual fields to the collective fields farmed by men on behalf of the household. Lilja, Sanders, Durham, De Groote, and Dembélé (1996) in Mali also found that the introduction of new technologies in cash crops grown on collective plots increased women’s compensation for labor on those fields, reducing the male-female wage differential. Applying a programming model representative of conditions in southwestern Burkina Faso, Lawrence, Sanders, and Ramaswamy (1999) concluded that the impact of adopting farm technologies (as compared to household technologies) on women depended on the type of intrahousehold decision-making process—and was more favorable with bargaining behavior.

Here we develop a conceptual model that illustrates how technology adoption is affected by intrahousehold bargaining and enables us to test econometrically the nature of the linkage between input use on collective and individual fields. We apply probit and tobit models to data collected during three cropping seasons (2009/10, 2010/11 and 2011/12) under the Continuous Farm Household Survey (*Enquête Permanente Agricole* (EPA) of Burkina Faso. We employ the Mundlak-Chamberlain device to address time-invariant unobserved effects that may be related to household decision-making. The significance, direction, and magnitude of the regression coefficients reveal information about the negotiations between the head who manages collective fields on behalf of the extended family and individuals who have been allocated plots to meet their personal needs.

Our findings have importance for development policy. When family resources are managed both individually and collectively, the relative bargaining position of family members affects the intended and unintended outcomes of policies and programs (Haddad & Kanbur, 1992; Jacoby, 2002, Smith and Chavas, 2007, Doss, 2013). For example, Smith and Chavas (1997) concluded that male-favored bargaining in Burkinabe households restricted the positive effects of rising income on the physical well-being of women. Here, using fertilizer as a case in point, we demonstrate how the diffusion of new technologies could be affected by the bargaining positions of household members. We highlight fertilizer adoption for two reasons. First, despite its low average use in Burkina Faso relative to other countries, fertilizer is fundamental for enhancing productivity and is the most widely adopted modern input. Second, fertilizer is a divisible input that can be readily allocated among plots. Our model can be easily adapted to the study of various intrahousehold bargaining processes in agricultural production, including husband-wife and intergenerational decision-making, and extended to other types of farm technology.

Next, we highlight pertinent contextual features of the farming system. The presentation of the theoretical model follows. Section 4 summarizes the empirical strategy. Results are discussed in Section 5, and conclusions are drawn in Section 6.

## 2 The Burkinabe farming context

Over two-thirds of the Burkinabe population depends on agriculture as their primary source of livelihood (World Bank, 2016). Hence, agricultural intensification is crucial for increasing household incomes. Production of rainfed cereals, such as sorghum, millet, and maize, account for over 70% of total cultivated land (INSD, 2014). Needing less moisture, millet and sorghum are well-adapted to drylands and are cultivated throughout the country. Both cereals play an important role in achieving food security, since they constitute the basis of the diet for a vast majority of Burkinabe (DGPER, 2012). In contrast, maize is grown only in the wetter zones of the country. Cotton, the main country’s export, is also produced in the wetter zones, where it is typically grown in rotation with maize and millet or sorghum. Households growing cotton have benefited for years from a vertically-integrated and highly institutionalized cotton sector, which provides them with fertilizer on credit for cotton and cereal crops (Theriault & Serra, 2014).

Social norms in most of Burkina Faso are patriarchal and patrilineal. The senior male head has ultimate responsibility for ensuring the household’s food security, supervising the use of household labor and inputs on the major collective fields planted to cereals and cotton. Harvests from collective fields are shared as meals consumed together by the patriarch, who ‘holds the keys’ to the family granaries and distributes their content. Sales revenues serve to purchase common goods, such as ceremonial expenses or taxes (Becker, 1996, West, 2010). Each household member contributes to labor on collective fields, and has a strong incentive to do so because the head is obliged to provide public goods in return (Kazianga & Wahhaj, 2013).

Alongside the collective field, the head may also allocate plots among individual members of the household according to both norms and negotiation. Following patrilocal norms, on marriage, women join the family of their husband and gain the right to cultivate a plot, on which they grow crops needed for food preparation. Among many ethnic groups in Burkina Faso, but not all, proceeds from these fields are hers “without encumbrance” (Kevane & Gray, 1999: 8). In addition to the married sons and their wives, unmarried sons and younger brothers of the head, as well as widows, may be allocated fields to supplement their personal needs. In times of duress when the family granaries are low, individuals may also be called upon to contribute to the common good from their individual proceeds (Thorsen, 2002, Van den Broek, 2009).

Use rights, as expressed in the right to manage production on an individual plot, are negotiable. Kevane and Gray (1999) describe how women’s indirect rights to the fields they obtain through marriage are mediated by the broader range of duties and responsibilities undertaken by both men and women in the household, and often linked directly to labor use. Household members are expected to supply labor to the collective fields before their own fields (Becker, 1990, van Koppen, 2009). Yet, young male plot managers interviewed by Guirkinger and Platteau (2014) admitted to prioritizing their individual fields. Guirkinger and Platteau (2014) also found that the numbers of individual plots allocated rose with the numbers of young married men in the extended family household. The allocation of individual fields to junior household members (e.g., women and young married men) is seen as a means of generating incentives to enhance labor productivity. Regardless of the household member, any individual plots could be re-allocated from one season to the next, giving the head more authority (Lawrence et al., 1999).

Customarily, land rights for the extended family household as a whole are vested in the head, following the ancestral rights of lineages as “first” cultivators or rights conferred to others, such as migrants, by the traditional land chief (Kevane & Gray, 1999). Despite the legislative efforts of the Burkinabe government to encourage titling, its incidence remains limited and is superseded by customary rules (Hughes, 2014). Land use remains the key strategy employed by households to strengthen their claim over land. A review of the literature on land rights and investment incentives in West Africa, including Burkina Faso, shows that formal tenure security has no effect on incentives to use modern inputs such as fertilizer but does affect fallow and tree planting (Fenske, 2011). In Burkina Faso, Brasselle, Gaspart, and Platteau (2002) found that no category of land rights has a significant impact on investment. Both studies conclude that customary tenure norms provide the basic land rights required to stimulate small-scale investment with shorter time horizons.

Fertilizer use is not, per se, an investment in the quality of the land unless continued over successive seasons. Fertilizer is an economic investment, however. Average use rates in Burkina Faso are low on a world scale. For instance, fertilizer consumption per hectare of arable land averages 11 kg in Burkina Faso, which seems high compared to other West African Sahel countries (e.g., 0.49 kg/ha in Niger; 5 kg/ha in Benin; 9 kg/ha in Nigeria) but very low compared to Eastern and Southern African countries (e.g., 35 kg/ha in Zambia; 38 kg/ha in Kenya; 58 kg/ha in South Africa) (World Bank, 2016). The government of Burkina Faso instituted a fertilizer subsidy in 2008 in an effort to stimulate use, particularly on maize and rice because these crops respond better to fertilizer than either sorghum or millet. Recipients of the subsidy, and any fertilizer received through official programs, are usually the heads of households. Official sources still dominate fertilizer supply in Burkina Faso because of the scarcity of fertilizer and underdevelopment of commercial markets. In the model we present below, we propose that intrahousehold distribution of fertilizer by the head is a means of incentivizing members; at the same time, members influence their bargaining position through agreeing to supply (or withhold) labor.

## 3 Theoretical model

We extend the intrahousehold decision-making model proposed by Kazianga and Wahhaj (2013) to understand the allocation of a modern, divisible input (fertilizer) between plots managed by the household head and those managed by other household members. We show how the intrinsic complementarity of fertilizer and labor inputs^2^ has implications for the bargaining positions of household members, and thus for policies and programs aimed at intensifying agriculture through fertilizer adoption in Burkina Faso.

We examine the simple case of a household composed of only a head (h) and a junior member (m). The model has similar implications when extended to multiple junior members and wives (derivations available from the authors). The household head and junior member each manages one plot of land (collective and individual fields, respectively), growing the same crop Y that has a price normalized to 1. The junior member has ${\text{E}}_{\text{l}}^{\text{m}}>0$ hours of productive labor endowment while the head has none of his own and must rely on labor from the junior member $\left({\text{E}}_{\text{l}}^{\text{h}}=0\right)$. Unlike labor, the household head has a positive fertilizer endowment $\left({\text{E}}_{\text{z}}^{\text{h}}>0\right)$, while the junior member does not $\left({\text{E}}_{\text{z}}^{\text{m}}=0\right)$. As explained in Section 2, these assumptions are based on a context where the household head plays more of a supervisory role and junior members provide most labor for production. Access to a scarce, costly input like fertilizer, and to the programs that promote its use, is generally the privilege and the responsibility of the senior head.

The common production technology uses labor (L) and fertilizer (Z) inputs to produce Y. Output also depends on plot characteristics (A), such as land quality. Since each individual has only one plot, the labor, fertilizer and plot characteristics can be subscripted by the member. Hence ${\text{Y}}^{\text{i}}=\text{F}({\text{L}}^{\text{i}}\text{,}{\text{Z}}^{\text{i}}\text{,}{\text{A}}^{\text{i}})$ for i=h,m, where F is a strictly concave function. Labor is more productive on plots where more fertilizer is applied. This complementarity in production has interesting and meaningful effects on the optimal allocation of inputs because it can influence bargaining positions among household members, as explained below.

On the consumption side, there is one private good x with price ${\text{p}}_{\text{x}}$, and one public good q with price ${\text{p}}_{\text{q}}$. The junior member is free to spend his income on x or q. However, the household head is compelled by social norms to spend *a* percent of his income on q. Departing from the model of Kazianga and Wahhaj (2013), who assumed that the head spends all the income from his plot on the public good, we assume that he spends a fixed proportion. The advantage of this approach is that while the junior member has an incentive to supply labor on the head’s plot, the head has a more active role in trying to increase his own output and allocating expenditure across the public and private goods. Utility is a function of the public and private good i.e. ${\text{U}}^{\text{i}}({\text{x}}^{\text{i}}\text{,q})$ for i=h,m. Since there are no savings in this model, the entire income is spent on the two goods.

### 3.1 Cooperative solution

First, we use the collective model, where λ denote the Pareto weights, to benchmark the efficient allocation of resources. Then, similar to Fafchamps (2001), we explore conditions under which an efficient allocation could be sustained voluntarily over time.

The collective model problem is:

$$\max_{x^{h}\text{,}x^{m}\text{,}q\text{,}L^{h}\text{,}Z^{h}\text{,}L^{m}\text{,}Z^{m}}U^{h}(x^{h}\text{,}q)+(1-\lambda )U^{m}(x^{m}\text{,}q)s.t\text{.}$$

$$p_{x}(x^{h}+x^{m})+p_{q}q⩽Y$$

$$\frac{p_{q}q}{Y^{h}}⩾a$$

$$Y=Y^{h}+Y^{m}=F(L^{h}\text{,}Z^{h}\text{,}A^{h})+F(L^{m}\text{,}Z^{m}\text{,}A^{m})$$

$$L^{h}+L^{m}⩽E_{L}^{m}$$

$$Z^{h}+Z^{m}⩽E_{z}^{h}$$

In the case that the public good constraint binds, a percentage of income is spent on the public good and the remaining is allocated between the private goods of the head and junior member to equate weighted marginal utilities. If the public good is highly desired by household members, a greater percentage is spent on the public good – up to the point where the per dollar marginal utility is equated between the public and private good.

Our particular concern is the production side. When production is Pareto-efficient, labor and fertilizer are allocated across the head and junior member’s plots to maximize production given a fixed endowment of inputs. Hence the marginal product of labor and marginal product of fertilizer is equated across plots $\left(\frac{\partial F(L^{h}\text{,}Z^{h}\text{,}A^{h})}{\partial L^{h}}=\frac{\partial F(L^{m}\text{,}Z^{m}\text{,}A^{m})}{\partial L^{m}}\;\text{and}\;\frac{\partial F(L^{h}\text{,}Z^{h}\text{,}A^{h})}{\partial Z^{h}}=\frac{\partial F(L^{m}\text{,}Z^{m}\text{,}A^{m})}{\partial Z^{m}}\right)$ An implication is that $Z^{h}=Z^{m}$ if $L^{h}=L^{m}$ and $A^{h}=A^{m}$. In other words, use of fertilizer is the same across plots if labor inputs and land characteristics are the same. In the empirical estimation, we will test whether fertilizer is applied equally across plots, conditional on both plots having similar plot characteristics and plot manager characteristics.

### 3.2 Non-cooperative solution

Next, we find the ‘autarky’ solution, which is defined as the allocation under which the head and junior member cannot achieve the efficient outcome because they are unable to commit resources (labor, fertilizer) to one another. The term ‘autarky’ is used loosely since the non-cooperative model will not necessarily result in an allocation in which both keep their own endowments; instead, the junior member may want to allocate labor to the head’s plot even if the head is not allocating fertilizer to the junior member’s plot. The household head, who in the local context is given the overall authority for farm production in the household, moves first. Also, in reality there are multiple junior members in most households which allows heads to negotiate with them and exert bargaining power.

The head’s problem is:

$$\max_{x_{h}^{h}\text{,}x_{h}^{m}\text{,}q^{h}\text{,}Z_{h}^{h}\text{,}Z_{h}^{m}}U^{h}(x_{h}^{h}+x_{m}^{h}\text{,}q^{h}+q^{m*})s.t\text{.}$$

$$p_{x}(x_{h}^{h}+x_{h}^{m})+p_{q}q^{h}⩽Y^{h}$$

$$\frac{p_{q}q^{h}}{Y^{h}}⩾a$$

$$Y^{h}=F(L_{m}^{h}\text{,}Z_{h}^{h}\text{,}A^{h})$$

$$Z_{h}^{h}+Z_{h}^{m}⩽E_{z}^{h}$$

while the junior member’s problem is:

$$\max_{x_{m}^{m}\text{,}x_{m}^{h}\text{,}q^{m}\text{,}L_{m}^{m}\text{,}L_{m}^{h}}U^{h}(x_{m}^{m}+x_{h}^{m}\text{,}q^{m}+q^{h*})s.t\text{.}$$

$$p_{x}(x_{m}^{m}+x_{m}^{h})+p_{q}q^{m}⩽Y^{m}$$

$$Y^{m}=F(L_{m}^{m}\text{,}Z_{h}^{m}\text{,}A^{m})$$

$$L_{m}^{m}+L_{m}^{h}⩽E_{L}^{m}$$

$q^{m*}$ and $q^{h*}$ refer to the optimal public good purchases of the junior member and the household head respectively. The head will allocate fertilizer across the plots so that $\frac{1}{p_{q}}\frac{\partial F}{\partial Z_{h}^{h}}+\frac{\partial q^{m*}}{\partial Z_{h}^{h}}=0$. The additional income from using an extra unit of fertilizer on the head’s plot, $\frac{\partial F}{\partial Z_{h}^{h}}$, can be used to purchase $\frac{1}{p_{q}}\frac{\partial F}{\partial Z_{h}^{h}}$ units of q. At the optimum, this will equal $\frac{\partial q^{m*}}{\partial Z_{h}^{h}}$, the loss in public good contributed by the junior member due to more fertilizer applied on the head’s plot (which leads to less fertilizer applied on the junior member’s plot). $\frac{\partial q^{m*}}{\partial Z_{h}^{h}}⩽0$ since the junior member is not required to contribute to the public good. In case the junior member does not purchase the public good, $\frac{\partial q^{m*}}{\partial Z_{h}^{h}}$ equals zero and the head will apply all the fertilizer to his own plot i.e. $Z_{h}^{h}=E_{z}^{h}$. This is because the head does not benefit from higher income on the junior member’s plot, since all of it is spent on the private good. Even if the junior member contributes to the public good, this contribution is likely to be low and the head will allocate a larger portion of fertilizer on his own plot rather than the junior member’s plot.

The junior member will allocate labor so that $\frac{1}{p_{q}}\frac{\partial F}{\partial L_{m}^{m}}+\frac{\partial q^{h*}}{\partial L_{m}^{m}}=0$. The additional income gained by the junior member from using an extra unit of labor on his plot, $\frac{\partial F}{\partial L_{m}^{m}}$, can be used to purchase $\frac{1}{p_{q}}\frac{\partial F}{\partial L_{m}^{m}}$ units of q. At the optimum, this will equal $\frac{\partial q^{h*}}{\partial L_{m}^{m}}$, the loss in public good contributed by the household head due to less labor applied to the head’s field. But $\frac{\partial q^{h*}}{\partial L_{m}^{m}}\neq 0$ rather $\frac{\partial q^{h*}}{\partial L_{m}^{m}}<0$, since the head is obliged to spend a share of the income from his plot on the public good due to social norms that he protects food security in the household.

While we expect more fertilizer to be allocated to the head’s plot than the junior member’s plot, we expect labor to be even more evenly distributed – more labor may actually be allocated to the collective plot—for two reasons. First, social norms act as a commitment mechanism for the head to spend a fixed proportion of income on the public good. Second, the marginal product of labor will be higher on the head’s plot due to higher application of fertilizer. Input complementarity in production enables the head to exert some control over labor allocation despite not having any labor endowment.

### 3.3 Repeated interaction

We now examine conditions under which the household can voluntarily achieve the efficient allocation of resources and emulate a cooperative solution through bargaining. Since there is repeated interaction over time between the head and the junior member, they can sustain the efficient solution in the collective model. From the second welfare theorem, we know that a Pareto efficient resource allocation can be represented as a price system combined with lump-sum transfers (Fafchamps, 2001).

Let $U_{c}^{h}$ and $U_{c}^{m}$ be the household head and junior member’s single period utility under the collective solution while $U_{a}^{h}$ and $U_{a}^{m}$ are defined as their single period autarky solution. The collective solution can be sustained under the following condition:

$$\overset{\infty }{\underset{t=0}{\sum }}\delta_{i}^{t}U_{c}^{i}>U_{s}^{i}+\overset{\infty }{\underset{t=1}{\sum }}\delta_{i}^{t}U_{a}^{i}\;\text{for}\;\text{i}=\text{h,m}$$

$U_{s}^{i}$ denotes the utility of i by unilaterally deviating from the collective model allocations. For the household head, $U_{s}^{h}$ is the solution to:

$$\max_{x_{h}^{h}\text{,}q^{h}\text{,}Z_{h}^{h}\text{,}Z_{h}^{m}}U^{h}(x_{h}^{h}+x_{m\text{,}c}^{h}\text{,}q^{h}+q_{c}^{m})s.t\text{.}$$

$$p_{x}(x_{h}^{h}+x_{h}^{m})+p_{q}q^{h}⩽F(L_{m\text{,}c}^{h}\text{,}Z_{h}^{h}\text{,}A^{h})$$

$$\frac{p_{q}q^{h}}{Y^{h}}⩾a$$

$$Z_{h}^{h}+Z_{h}^{m}⩽E_{z}^{h}$$

where $x_{m\text{,}c}^{h}$ and $q_{c}^{m}$ are the collective model allocations chosen by the junior member.

For the junior member, $U_{s}^{m}$ is the solution to:

$$\max_{x_{m}^{m}\text{,}q^{m}\text{,}L_{m}^{m}\text{,}L_{m}^{h}}U^{h}(x_{m}^{m}+x_{h\text{,}c}^{m}\text{,}q^{m}+q_{c}^{h})s.t\text{.}$$

$$p_{x}(x_{m}^{m}+x_{m}^{h})+p_{q}q^{m}⩽F(L_{m}^{m}\text{,}Z_{h\text{,}c}^{m}\text{,}A^{m})$$

$$L_{m}^{m}+L_{m}^{h}⩽E_{L}^{m}$$

where $x_{h\text{,}c}^{m}$ and $q_{c}^{h}$ are the collective model allocations chosen by the head.

This suggests that the collective model solution can be sustained if the discounted lifetime utility from cooperating is forever higher than utility of deviating one period and then receiving the autarky utility.

## 4 Empirical strategy

The model predicts that if allocation of inputs is efficient, fertilizer use per hectare will be equalized conditional on observable characteristics of plots or managers that might affect marginal productivity of fertilizer. If household members can cooperate and agree to this efficient allocation of inputs, an additional kg per ha of fertilizer on the collective plot will lead to one more kg per ha of fertilizer being applied to the individual fields. In the reduced form (*individual fertilizer* = *f(crop, collective fertilizer, plot manager characteristics, plot characteristics, household characteristics, market characteristics, and weather)*, we will expect *collective fertilizer* to have a coefficient close to one when this efficient allocation of inputs is achieved.

However, if the household is unable to cooperate and sustain the efficient allocation of inputs, we predict that the coefficient on *collective fertilizer* will be less than one. If the coefficient on *collective fertilizer* is not significantly different from zero, the solution resembles autarky, with an absence of commitment among members. This is because the household head applies all the fertilizer on his own plot, and does not apply any to the junior member’s plot. A coefficient on *collective fertilizer* that is significantly different from zero, but less than one, suggests a bargaining outcome that is inefficient. This is because while the household head shares fertilizer with junior members, he shares less than what is optimal. The conceptual model shows that the timing of the fertilizer allocation decision matters – if the household head commits a large amount of fertilizer to his own plot, he can influence junior members and make them provide more labor to the collective plot. The household head will first allocate fertilizer to his own plot, and then the remaining fertilizer and labor will be allocated across plots. The initial allocation of a large amount of fertilizer on his own field will oblige the junior member to allocate more labor to his collective field in an effort to secure more of the scarce, purchased input. In this case, we would observe unequal distribution of fertilizer across plots; every additional kg of fertilizer applied to the collective field would lead to less than one kg of fertilizer applied to the individual fields. We interpret a larger, positive marginal effect of collective use on individual use as the ability of household members to cooperate toward and sustain an efficient allocation of inputs.

### 4.1 Econometric approach

We use both a seemingly unrelated, bivariate probit model and a recursive formulation to test the nature of the relationship between the decision to use fertilizer on collectively-managed fields and on individually-managed fields. Following Maddala (1986), the dependent variables in the regression system represent latent variables for which only the dichotomous outcomes are observed.

We can represent the decisions to apply fertilizer to collectively managed and individually managed plots by the unobserved latent variables model:

$$y_{1}^{*}=x_{1}\beta_{1}+e_{1}$$

$$y_{2}^{*}=x_{2}\beta_{2}+e_{2}$$

where ${\text{y}}_{1}^{*}$ is the underlying profitability of using fertilizer on collectively managed plots while ${\text{y}}_{2}^{*}$ is the underlying profitability of using fertilizer on individually managed plots. ${\text{x}}_{1}$ and ${\text{x}}_{2}$ are the set of variables that explain the utility of income from fertilizer use on collectively and individually managed plots.

The bivariate probit model defines the outcomes as:

$$y_{1}=1\;\text{if}\;y_{1}^{*}>0\text{,}=0\;\mathrm{otherwise}$$

$$y_{2}=1\;\text{if}\;y_{2}^{*}>0\text{,}=0\;\mathrm{otherwise}$$

and

$$\left(\begin{array}{c} \varepsilon_{1}\\ \varepsilon_{2} \end{array}\right)|X∼N\left(\begin{array}{c} 0\\ 0 \end{array}\text{,}\left[\begin{array}{cc} 1 & \rho \\ \rho & 1 \end{array}\right]\right)$$

${\text{y}}_{1}$ indicates whether fertilizer was applied to a collectively managed plot and ${\text{y}}_{2}$ indicates whether fertilizer was applied to an individually managed plot.

The seemingly-unrelated, bivariate probit specification addresses potential simultaneity by taking into account the correlation between the residuals of the two equations in the system. The appropriateness of the bivariate model as compared with the separate probit models can be evaluated with a likelihood ratio test. The independent univariate models are nested within the multi-equation model, which represents the unconstrained regression. The Wald test indicates whether the error structures are related, as represented by the estimated correlation coefficient ρ-hat. Failure to reject the null hypothesis that *ρ* equals zero leads to separate estimation of the probit equations.

The recursive formulation introduces the potential for a causality in one direction. In addition to a shared vector of exogenous variables in each pair of equations, the fertilizer use equation for individually-managed plots includes the binary variable indicating use of fertilizer on collectively-managed plots (Z). In our recursive formulation, we posit that decisions on collectively-managed fields supersede or precede those taken on individually-managed fields according to the goal of ‘family welfare first.’ Generally, in this social organization of production, access to inputs, including fertilizer, accrues initially to the patriarchal decision-maker or team leader who is responsible for ensuring household food security. The statistical test on the coefficient of the regressor (*Z*), which indicates fertilizer use on collectively-managed fields, would provide evidence of a systematic relationship. According to Wilde (2000), in contrast to linear simultaneous equations with only continuous endogenous variables, in recursive multiple equation probit models with endogenous dummy regressors, no exclusion restrictions for the exogenous variables are needed if there is sufficient variation in the data. That condition is ensured by the assumption that each equation contains at least one varying exogenous regressor, although this is an assumption that is rather weak in economic applications. However, we also address the exclusion restriction by including plot characteristics in the equations, which differ for individually and collectively managed plots.

We also wish to test whether application rates for fertilizer on collective plots are related to application rates of fertilizer on individual plots. Since a significant proportion of the sample does not use any fertilizer, OLS is not appropriate. Hence, we estimate a tobit regression to test this relationship for nitrogen nutrient kg per hectare on individually and collectively managed plots.

The fertilizer use (nitrogen nutrient kg per hectare) model is represented by:

$$y_{1}=x_{1}\beta_{1}+e_{1}$$

$$y_{2}=\alpha_{1}y_{1}+x_{2}\beta_{2}+e_{2}$$

where $y_{1}$ is the nitrogen nutrient kg per hectare on collectively managed plots while $y_{2}$ is the nitrogen nutrient kg per hectare on individually managed plots. ${\text{x}}_{1}$ and ${\text{x}}_{2}$ are the set of variables that explain fertilizer use on collectively and individually managed plots.

We first test whether $y_{1}$ is endogenous in the equation for $y_{2}$ using a control function approach (CFA). Collective plot characteristics are included in $x_{1}$ but not in $x_{2}$, hence they are the instruments used for identification. If we fail to find evidence that $y_{1}$ is endogenous in the equation for $y_{2}$, we can estimate the equation for $y_{2}$ directly and obtain consistent estimates of $\alpha_{1}$. If we find evidence of endogeneity, we will rely on the control function approach for consistent estimates of $\alpha_{1}$.

In each of our nonlinear models, we also employ the Mundlak-Chamberlain (also known as the correlated random effects-CRE) device to address time-invariant unobserved effects that may be related to household decision-making. An advantage of random effects models is that time-invariant observed variables, which are dropped when applying fixed effects models, can be retained in the regression. However, these models have the requirement that individual-specific effects be uncorrelated with the explanatory variables. The Mundlak-Chamberlain approach corrects for possible violation of the independence assumption between the covariates and the error term in the random effects model. Recommended for nonlinear models, this technique involves including the means of variables constructed at the household level that vary over time. Also, we do not have a panel at the plot level, which limits our ability to control for plot-level unobserved variables.

### 4.2 Data

We utilize data from the Continuous Farm Household Survey (*Enquête Permanente Agricole* (EPA)) of Burkina Faso, which are collected by the General Research and Sectoral Statistics Department (*Direction Générale des Études et des Statistiques Sectorielles* (DGESS)) of the Ministry of Agriculture and Food Security (*Ministère de l’Agriculture et de la Sécurité alimentaire* (MASA)). The sampling frame for the EPA is based on the 2006 Population Census, and consists of 4130 household farms in 826 villages across all 45 provinces. The EPA is used to estimate farm input use, production, area and yield of rainfed crops, and provides information about livestock holdings, income and expenditures of rural households. In this analysis, we utilize data for 2009/10, 2010/11 and 2011/12 cropping seasons (three survey years). These are the last years for which fully cleaned data are available.

After eliminating households that were not continuously surveyed throughout the three-year period and missing observations for variables of interest, we are left with over 2700 households. We estimate the model with maize plots only because fertilizer use is under 10% on other major cereals crops (sorghum, millet) in the seasons studied. Including these crops reduces the explanatory power of the regressions considerably.

Many households cultivate multiple collective plots of maize (9321 total collective plots), and only some cultivate individual plots of maize (1475 individual plots). For our two-stage tobit model, in order to match observations, we took only the largest collective maize field and all individual plots that were not managed by the head. We excluded plots on which maize was not the primary crop. The first-stage observations include 5802 collective maize fields, while the second-stage includes matched sets of 556 collective and individual maize fields. The bivariate probit model includes 506 observations, considering some missing observations among independent variables.

Rainfall estimates from the National Oceanic and Atmospheric Administration’s Climate Prediction Center at the commune level are used to control for rainfall variability.

### 4.3 Variables

Exogenous explanatory variables are operationally defined and summarized in Table 1. Plot manager characteristics include the gender and age, which affect the status of household members, and literacy and membership or leadership in farmer associations of any type (formal or informal, any crop or service focus), which may enhance bargaining skills. Gender and age are highly correlated with marital status. Receipt of credit within the 12 months preceding the survey is strongly and significantly correlated with membership. We observe that only 4 percent of collective plots are managed by females, as compared to 50 percent of individual plots. Overall, women manage about 8 percent of the maize plots in our analytical sample. As expected, collective plot managers are considerably older on average (50 years) compared with individual plot managers (36 years). Membership in any farmer organization, and particularly a leadership role, is much more frequent among managers of collective plots, and the number of years since the last extension visit are significantly fewer. In fact, all characteristics except literacy rates differ significantly between plot manager categories. We use literacy rather than primary education because of the low rates of public education overall. About one quarter of plot managers are literate.

**Table 1:** Definitions and descriptive statistics of explanatory variables.

		(1)	(2)	(3)	(4)
		All	Collectively Managed	Individually Managed	p-Value (2)-(3) = 0
			Mean (SD)		
Plot manager is female	If the plot manager is female = 1; Otherwise = 0	0.0793 (0.270)	0.0369 (0.189)	0.496 (0.500)	0.000
Plot manager is literate	If the plot manager is literate = 1; Otherwise = 0	0.237 (0.426)	0.239 (0.426)	0.226 (0.418)	0.494
Age of plot manager	Age of the plot manager	48.6 (14.9)	49.8 (14.3)	36.2 (14.9)	0.000
Plot manager is FO leader	If the plot manager is leader of a farmer organization = 1; Otherwise = 0	0.107 (0.309)	0.114 (0.318)	0.0342 (0.182)	0.000
Plot manager is FO member	If the plot manager is a member of a farmer organization = 1; Otherwise = 0	0.191 (0.393)	0.201 (0.401)	0.0935 (0.291)	0.000
Years since contact with extension	Number of years since the plot manager has received any extension services (yrs)	4.68 (0.960)	4.66 (0.988)	4.90 (0.584)	0.000
Plot sloped	If the plot is sloped = 1; Otherwise = 0	0.0651 (0.247)	0.0651 (0.247)	0.0647 (0.246)	0.972
Plot far from home	If the plot is far from the house = 1; Otherwise = 0	0.377 (0.485)	0.364 (0.481)	0.511 (0.500)	0.000
Plot in lowlands	If it is a lowland plot = 1 Otherwise = 0	0.0505 (0.219)	0.0435 (0.204)	0.119 (0.324)	0.000
Plot size	Size of the plot (hectares)	0.589 (1.03)	0.615 (1.07)	0.326 (0.443)	0.000
No. of years since plot last fallowed	Number of years since the plot was last left fallow	18.75 (14.9)	19.6 (15.0)	10.6 (11.0)	0.000
Tree exists on plot	Trees growing on the plot	0.601 (0.490)	0.600 (0.490)	0.610 (0.488)	0.643
No. of adults in household per hectare	Number of adults in the household divided by household area cultivated (ha)	3.03 (9.10)	3.09 (9.50)	2.42 (2.90)	0.047
Non-farm income	Value of non-farm income at the household level (ln 000 CFA)	2.93 (2.49)	2.89 (2.47)	3.32 (2.67)	0.000
Livestock (TLU)	Amount of livestock owned by the household (TLU)	7.69 (17.3)	7.31 (17.4)	11.4 (16.1)	0.000
No. of agro-dealers	Number of agro-dealers in each province (units)	31.9 (32.4)	32.4 (32.9)	26.5 (26.1)	0.000
Province population density	Province population divided by area (km^2^)	82.8 (120.2)	84.9 (123.7)	61.8 (74. 9)	0.000
Rainfall	Annual rainfall in each commune (mm)	888.6 (185.3)	885.3 (185.6)	921.8 (179.7)	0.000

*Source:* Author calculations from EPA.

Plot characteristics include whether the plot is located far from the house (a day trip to the “brousse” or overnight trip – “campement”—as compared to the plots that are adjacent to the house), and whether the plot is located in the lowlands or is sloped. The size of the plot controls for scale, and tests the hypothesis that fertilizer use and rates of use are neutral to scale. Fertilizer is generally considered to be neutral to scale—meaning that its rate of use would not depend on the size of the field or farm. The data demonstrate that on average, collectively-managed plots are nearly twice the size of individually-managed maize plots (0.62 v. 0.33 ha, respectively), and slightly (but significantly) less likely to be found in the lowlands. In addition to these variables, we control for the number of years since the plot was last fallowed and whether the plot has agroforestry. Both are indicators of land quality, and are also hypothesized to affect demand for fertilizer as an economic investment. Interestingly, 60% of plots, regardless of management type, have trees. The length of the time since fallow is twice as long for collectively-managed plots compared with individually-managed plots. At 20 years since fallowing on average for the family maize fields, the land quality problems that drive the need for use of fertilizer, manure, and other soil and water conservation practices are evident.

Household covariates are included primarily to control for time-invariant unobserved heterogeneity, as explained above. Our model suggests that the number of adults in the household, per hectare, could affect the bargaining position of any single member. The data suggest an average of 3 adults per hectare, although households that also have individual maize plots have significantly fewer adults per hectare—more land per working adult. On the other hand, these households also have more non-farm income, which could relieve liquidity and credit constraints. They also have considerably more livestock, which provides manure and serves as a sign of wealth.

As measures of market infrastructure, we employ the number of agro-dealers in the province and the overall population density. Households with individual maize plots are located in provinces with fewer agro-dealers but lower population densities. The rainfall variable is constructed as the coefficients of variation in total annual rainfall at the commune level over the last three years, which we consider the pertinent decision-making period for farmers because it is recent in their memories. Households with both individual and collective plots are located in communes with higher average coefficients of variation in recent years.

## 5 Findings

### 5.1 Descriptive statistics

The data reveal that labor is indeed traded within the household. About 80 percent of plots across years receive labor input from one or more household members (up to over 6 different members, but most often, in 37% of cases, a single additional member) apart for the plot manager him/herself. Another reason for high amounts of labor sharing is that some members have comparative advantage in certain tasks; men may be more efficient in clearing land since it requires more physical strength while women and children may be more effective at weeding and harvesting (Fafchamps, 2001, Prasad and Ram, 1990).

Such division of tasks across members indicates that households seek to allocate inputs with the aim of improving productive efficiency, and are not composed of members that are producing under autarky. Even though fertilizer-sharing transactions within the household are not recorded in the data, we contend that similarly to land and labor, it is likely that fertilizer is transacted within the household to increase allocative efficiency in production. However, in the analytical sample, 15 percent of households chose to apply fertilizer to the collective field but not to the individual plot, consistent with inability to reach a cooperative solution.

Overall, 41% of collective maize plots managed by the head received fertilizer and 42% of individual maize plots not managed by the head were fertilized. Unconditional mean rates of use appear to be slightly higher on the small plots managed individually than on collectively-managed maize plots, although the difference is not statistically significant (Table 2). These are twice as high as the average for all crops in Burkina Faso (see introduction). Mean application rates (unconditional and conditional) are similar on both types of plots, supporting the notion of a Pareto-efficient solution. At first glance, this finding contrasts with that reported by Guirkinger et al. (2015), although their data aggregated expenditures on chemical inputs. Research by Udry (1996) which was based on ICRISAT data from the 1980s, did not include mineral fertilizer.

**Table 2:** Rates of fertilizer use (nitrogen kg/ha).

	(1)	(2)	(3)
	Collectively Managed	Individually Managed	p-Value (2)–(3) = 0
		Mean (SD)	
Unconditional Mean (kg/ha = 0 or kg/ha > 0)			
Collectively Managed	20.075	20.943	
Individually Managed	(1.557)	(1.864)	0.430
Conditional Mean (kg/ha > 0)			
Collectively Managed	49.464	49.341	
Individually Managed	(3.751)	(3.655)	0.496

Source: Author calculations from EPA.

**Fig. 1:**
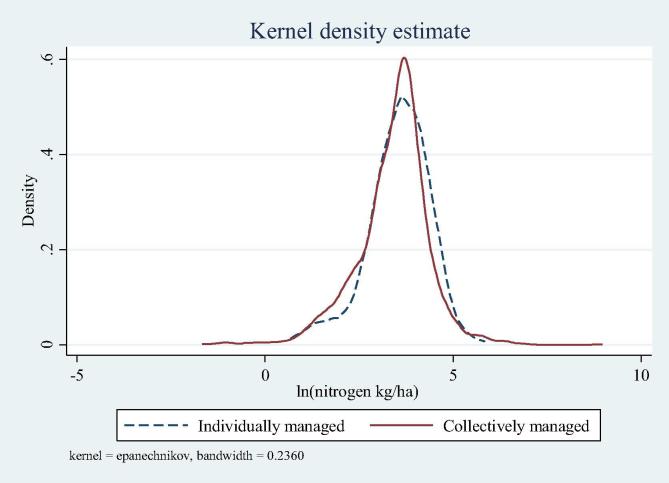
Distributions of fertilizer use rates on collectively and individually-managed plots.

Full distributions of values for positive use rates of nitrogen on maize plots managed collectively by the head and individually by other household members are shown in Fig. 1 (data in logarithms). The Kolmogorov-Smirnov test supports differences in underlying distributions with a p-value of 0.055, with a perceptible shift of the density to higher values on individual plots.

Descriptive statistics in Table 2 and Fig. 1 are based on bivariate statistics alone. Next, we report multivariate statistics based on the application of the theoretical framework.

### 5.2 Regression results

Average partial effects (APEs) from the seemingly-unrelated, bivariate probit regressions for individually- and collectively-managed plots are shown in Table 3, columns 1 and 2. We fail to reject the null hypothesis that the two equations are uncorrelated with a Wald test (p-value = .685). Thus, we estimate the final recursive models as two independent probit models.

**Table 3:** Bivariate probit (CRE) regressions for fertilizer use, individual and collective plots.

	Independent	Recursive model
	Individual plots (1)	Collective plots (2)
	APE	
Fertilizer used on collective plot	0.327***	
	(0.0282)	
Female plot manager	−0.0171	−0.00677
	(0.0374)	(0.0318)
Plot manager is literate	0.0297	0.0493***
	(0.0425)	(0.0139)
Age of plot manager	−0.00218	−0.000991**
	(0.00139)	(0.000442)
Plot manager is FO leader	0.141*	0.112***
	(0.0851)	(0.0190)
Plot manager is FO member	0.00366	0.171***
	(0.0589)	(0.0151)
Years since contact with extension	0.00317	−0.0305***
	(0.0285)	(0.00658)
Plot is sloped	0.0231	−0.00972
	(0.0662)	(0.0231)
Plot far from home	0.105***	0.144***
	(0.0377)	(0.0128)
Plot in lowlands	−0.134**	−0.0448
	(0.0528)	(0.0275)
Plot size	0.155***	0.124***
	(0.0504)	(0.00848)
No. of years since plot last fallowed	0.00224	−0.000677
	(0.00154)	(0.000423)
Tree exists on plot	0.0947**	0.0969***
	(0.0384)	(0.0128)
Adults in household per hectare	0.0108	−0.000854
	(0.00700)	(0.00104)
Non-farm income	−0.0383***	0.00211
	(0.0117)	(0.00385)
Livestock (TLU)	−0.00881***	−0.000445
	(0.00319)	(0.00118)
No of agro-dealers	0.00110	0.000862***
	(0.000731)	(0.000204)
Province population density	−2.47e−05	−0.000243***
	(0.000254)	(5.93e−05)
Rainfall	0.000185*	0.000153***
	(0.000106)	(3.82e−05)
Number of observations	517	5280

Robust standard errors in parentheses. N = 506. Year effects, constant term and means of time-varying household variables not reported. ^***^p < .01; ^**^p < .05; ^*^p < .10.

We find a positive, statistically significant coefficient that systematically relates the likelihood of fertilizer use on collectively-managed to fertilize use on individually-managed plots. The effect is large; adoption of fertilizer on a collective plot contributes to an average of a 0.33 rise in adoption probability on an individual plot. Thus, we find evidence that fertilizer is shared within the household.

Next, we explore the linkages in fertilizer use intensity. The results from the Tobit model (using the control function approach) are shown in Table 4. The residual from the first stage regression is statistically significant, leading us to conclude that fertilizer use intensity on collective fields is an endogenous regressor in the fertilizer use model for individual fields (column 2). Including the residual as a regressor allows us to find consistent estimates of the marginal effect of fertilizer use on the collective plots. We find that the marginal effect of an additional fertilizer kg/ha applied to collective fields on use rates applied to individual fields is 0.276 nitrogen nutrient kgs/ha (column 3). This is considerably lower than the efficient amount – we would expect this coefficient to be close to one if the efficient input allocations could be sustained.

**Table 4:** Tobit CFA (CRE) regressions for fertilizer use on individual plots.

	Collective plots (1)	Individual plots (2)	Individual plots (APE) (3)
Residual		−0.450*	
		(0.233)	
Nitrogen (kg/ha) on collective plot		0.722**	0.276**
		(0.323)	(0.120)
Female plot manager	61.02	−4.326	−1.654
	(64.15)	(8.512)	(3.283)
Plot manager is literate	15.26*	17.30*	6.618*
	(8.090)	(10.28)	(3.832)
Age of plot manager	−0.498*	−0.397	−0.152
	(0.282)	(0.333)	(0.127)
Plot manager is FO leader	46.93***	11.14	4.260
	(17.01)	(16.67)	(6.322)
Plot manager is FO member	59.39***	0.443	0.170
	(20.91)	(11.82)	(4.530)
Years since contact with extension	−8.064**	−4.027	−1.540
	(3.622)	(5.758)	(2.194)
Plot is sloped	−11.43	25.46	9.737
	(12.11)	(25.43)	(9.683)
Plot far from home	61.01***	18.70*	7.152*
	(20.48)	(11.31)	(4.310)
Plot in lowlands	−14.36	−24.90**	−9.522**
	(12.48)	(12.64)	(4.723)
Plot size	17.93**	−2.740	−1.048
	(8.896)	(8.323)	(3.154)
No if years since plot last fallowed	−0.428	0.388	0.149
	(0.271)	(0.346)	(0.132)
Tree exists on plot	44.25***	33.39***	12.77***
	(15.26)	(11.65)	(4.294)
Adults in household per hectare	0.168	2.211	0.846
	(0.784)	(3.923)	(1.492)
Non-farm income	0.891	−4.810	−1.839
	(1.270)	(3.039)	(1.147)
Livestock (TLU)	0.168	−2.341**	−0.895**
	(0.434)	(1.027)	(0.376)
No of agro-dealers	0.299***	0.121	0.0463
	(0.101)	(0.188)	(0.0705)
Province population density	−0.158**	−0.0286	−0.0109
	(0.0689)	(0.304)	(0.115)
Rainfall	0.0564**	0.0300	0.0115
	(0.0271)	(0.0233)	(0.00881)
Number of observations	5802	556	556

*Notes:* Bootstrapped standard errors in parentheses. Year effects, constant term and means of time-varying household variables not reported. ^***^p < .01; ^**^p < .05; ^*^p < 0.10.

The empirical strategy assumes that the collective and individual plots are similar after controlling for observable plot and plot manager characteristics as well as time-invariant household characteristics through the use the correlated random effects model. Hence, the same quantity of fertilizer per ha should be applied to them. Although some unobservable differences across these plots may not be captured, they are unlikely to explain such a disproportionate allocation of fertilizer—for every one nitrogen nutrient (kg/ha) applied on the collective field, only 0.276 kg/ha is applied to the individual field. This disproportionate allocation of fertilizer across plots is strong evidence of Pareto-inefficient allocation of inputs within the household.

Aside from the main tests of hypotheses, the bivariate probit results suggest that the determinants of fertilizer adoption also differ between collective plots managed by the head and individual plots managed by other household members (Table 3). Other than leadership in a farmer organization, none of plot manager characteristics (age, gender, literacy) has a statistically significant influence on likelihood of fertilizer use on individual plots. Evidently, household members can expect to share in use of fertilizer, independent of their status within the household. By contrast, literacy has a strong positive effect on the likelihood of fertilizer use collective plots, as does leadership and membership in farmer organizations. Older age reduces the likelihood that the manager of a collective plot uses fertilizer. The length of time since contact with an extension agent has a significant negative effect on the likelihood of fertilizer application to collective fields, but no discernible linkage to individual plots.

Plot characteristics affect the likelihood of use similarly across collective and individual plots. The presence of trees on the plot also has a large and significant effect overall–consistent with the findings of Fenske, 2011, Brasselle et al., 2002. The length of time since fallowing does not. However, Fenske (2011) notes that years of continuous cultivation is subject to recall bias and measurement error. Further, Matlon (1994) reported as long ago as 1994 that reliance on bush-fallow rotation was no longer feasible in the ICRISAT study villages. The average number of years since fallowing in our data confirm this point. The further from the home, the higher the probability that fertilizer is used—perhaps because these are newer fields with better fertilizer response. Larger plots have higher likelihoods of fertilizer use, regardless of plot management type. The density of agro-dealers in the area is positively and significantly related to the likelihood of fertilizer use on collective fields managed by the head—suggesting that fertilizer availability is indeed a constraint to adoption (Koussoubé & Nauges, 2016).

The literacy of the plot manager does appear to affect bargaining about the amounts of fertilizer to apply per hectare (Table 4). In terms of plot characteristics, results are similar for the intensity and likelihood of use. All plots farther away are more heavily fertilized; individual plots in the lowlands receive less fertilizer. The practice of agroforestry is in strong synergy with fertilizer adoption across plot management types. Similarly, controlling for population density, the number of agro-dealers in the province positively influences rates of fertilizer application as well as likelihood of use on plots managed by the head.

The statistical significance of plot size contradicts the assumption of scale-neutrality in fertilizer application, implying that larger collective fields receive not only larger total amounts but higher application rates (kg per ha). This finding may reflect the favorable bargaining position of heads who control larger land sizes and are able to garner more of this scarce resource relative to others. Scale bias among larger collective plots can be a consequence of differential access and lower transactions costs in a credit- or input-constrained environment (Feder, 1982).

## 6 Conclusions

We develop an intrahousehold model to explain the linkages between fertilizer adoption decisions on plots that are collectively managed by the head of the household and those that are individually managed by other household members. In the harsh agricultural environment of rural Burkina Faso, social norms guide the expectation that senior male heads will assume foremost responsibility for food security and other public goods needed for the survival of extended family households. We draw on this, and on the intrinsic complementarity of labor and fertilizer as divisible inputs, to illustrate how the introduction of a new modern input can influence bargaining outcomes. We test the model empirically by applying econometric models to nationally-representative panel data from Burkina Faso.

Descriptive statistics provide some evidence that household members seek to share and reach a Pareto-efficient, cooperative solution. Mean fertilizer rates are similar between plots managed by the head and those managed other household members, though when we consider the full range of observations, distributions are significantly different. Controlling for other factors, such as plot and plot manager characteristics, the econometric analysis demonstrates that the linkage between rates of use on collectively-managed and individually-managed plots is significant and positive but closer to zero than to one in magnitude. Given the theoretical framework, a coefficient of this size suggests that household members bargain for use of inputs by committing resources to one another, but do not attain the solution that emulates efficiency in a collective household. Moreover, the significance of plot manager characteristics that influence bargaining power, such as literacy, gender, age, contact with extension, and membership in farmer organizations differ between collectively- and individually-managed plots. The result confirms the differential status of household members in technology adoption. At the same time, regardless of plot manager, we find very strong positive effects of agroforestry on fertilizer use, which is encouraging and could be related to tenure security (Brasselle et al., 2002, Fenske, 2011, Goldstein and Udry, 2008).

We contribute to the literature on the intrahousehold decision-making by developing a theoretical model that allows us to examine the nature of the linkage between technology adoption on collective and individual plots as well as to test the efficiency of input use. Our hypothesis tests indicate that while there is input sharing occurring within extended family households in rural Burkina Faso, there is not enough bargaining to sustain the efficient allocation of inputs. The finding of inefficient resource allocation within households echoes that of previous research conducted with ICRISAT data collected during the 1980s in Burkina Faso (Kazianga and Wahhaj, 2013, Udry, 1996) and recent research in neighboring Mali (Guirkinger et al., 2015).

The supply of fertilizer to households is constrained in Burkina Faso (Koussoubé & Nauges, 2016). On one hand, findings suggest that an increase will “trickle down” to individuals within the households, and may explain why so many agricultural development programs and policies target household heads. Doing so is probably more cost-effective. However, inputs may not be equally distributed among household members and, thereby, among plots. If social welfare or efficient production, as compared to cost-effectiveness, is the most desired outcome, then designing inclusive problems may be more appropriate. For instance, if reducing youth rural-urban migration (to retain an optimal mix of labor in rural areas) through increasing agricultural productivity and ultimately income is the objective, then programs and policies to improved fertilizer access should ensure that young male and female managers of individual plots are included. With low rates of mechanization, an increase in modern input use is likely to lead to higher labor demand in rural areas, especially at peak times of the agricultural season. This may reduce the pressure on the urban economy to provide jobs for migrants and may lead to greater seasonal migration rather than permanent migration.

The design and implementation of effective agricultural programs and policy depends on a better understanding of the decision-making within households. There is an overall consensus in the literature that women are more efficient in utilizing cash transfer funds than men (see Fiszbein & Schady, 2009, for a review) and, therefore, virtually all conditional cash transfer programs target females as recipients. Such targeting, however, does not exist in programs that aim to increase agricultural input use. Directly targeting women to receive input subsidies or vouchers could lead to a more efficient use of inputs within the household. Indeed, our results suggest that agricultural output could increase if more inputs were allocated to the plots of junior members rather than the household head. Additionally, we may expect increased income of women to contribute more to the household’s welfare than if this extra income was earned by men (Fiszbein & Schady, 2009). A recent study by Theriault, Smale, and Haider (2017) shows that the likelihood of fertilizer adoption is significantly lower for female plot managers than their male counterparts in Burkina Faso. Therefore, greater access to inputs will improve the intrahousehold bargaining position of women while reducing the gender gap in technology adoption. Targeted programs have the potential to increase both efficiency and equity within household members, including not only gender but generational representation.

Our findings, though grounded in the particular socioeconomic context of Burkina Faso, have implications similar farming systems in West Africa. While we have analyzed fertilizer use on maize plots only – due to extremely low rates of fertilizer adoption among other cereals grown in Burkina Faso—tests of similar hypotheses with other crops would provide additional insights. The model developed here could be generalized more broadly to analyze the effects intrahousehold decision-making on technology diffusion within other household structures, between husbands and wives or among generations. A natural extension to our model would be to consider the introduction of a non-divisible input, such as anti-erosion, soil or water conservation structures, which underpin any efforts to sustainably intensify cereal production in the drier areas of the West Africa.

## Conflict of interest statement

On behalf of myself and my authors, I hereby state that there is no financial or personal interest or belief that could affect our objectivity in conducting and presenting this analysis.

## Footnotes

1 Another assumption that would be consistent with a unitary model would be a dictatorial welfare function.

2 Fertilizer is a labor-intensive modern input. The response of yield to fertilizer is enhanced by skilled, timely applications. If the price of fertilizer rises, we can expect less use of labor in fertilizer application; if wages rise, we can expect less use of fertilizer.
